# B Lymphocyte–Derived CCL7 Augments Neutrophil and Monocyte Recruitment, Exacerbating Acute Kidney Injury

**DOI:** 10.4049/jimmunol.2000454

**Published:** 2020-07-31

**Authors:** Akimichi Inaba, Zewen K. Tuong, Alexandra M. Riding, Rebeccah J. Mathews, Jack L. Martin, Kourosh Saeb-Parsy, Menna R. Clatworthy

**Affiliations:** * Molecular Immunity Unit, Department of Medicine, University of Cambridge, Cambridge CB2 0QH, United Kingdom;; † Cambridge University Hospitals National Health Service Foundation Trust, Cambridge CB2 0QQ, United Kingdom;; ‡ Cellular Genetics, Wellcome Trust Sanger Institute, Hinxton CB10 1RQ, United Kingdom;; § Department of Surgery, University of Cambridge, Cambridge CB2 0QQ, United Kingdom; and; ¶ National Institute for Health Research Cambridge Biomedical Research Centre, Cambridge CB2 0QQ, United Kingdom

## Abstract

Acute kidney injury (AKI) is a serious condition affecting one fifth of hospital inpatients. B lymphocytes have immunological functions beyond Ab production and may produce cytokines and chemokines that modulate inflammation. In this study, we investigated leukocyte responses in a mouse model of AKI and observed an increase in circulating and kidney B cells, particularly a B220^low^ subset, following AKI. We found that B cells produce the chemokine CCL7, with the potential to facilitate neutrophil and monocyte recruitment to the injured kidney. Siglec-G–deficient mice, which have increased numbers of B220^low^ innate B cells and a lower B cell activation threshold, had increased *Ccl7* transcripts, increased neutrophil and monocyte numbers in the kidney, and more severe AKI. CCL7 blockade in mice reduced myeloid cell infiltration into the kidney and ameliorated AKI. In two independent cohorts of human patients with AKI, we observed significantly higher *CCL7* transcripts compared with controls, and in a third cohort, we observed an increase in urinary CCL7 levels in AKI, supporting the clinical importance of this pathway. Together, our data suggest that B cells contribute to early sterile inflammation in AKI via the production of leukocyte-recruiting chemokines.

## Key Points

- AKI is a serious condition affecting one fifth of hospital patients.
- In AKI, B cells produce CCL7 and facilitate neutrophil and monocyte recruitment.
- CCL7 blockade in mice reduces myeloid cell infiltration and ameliorates AKI.

## Introduction

Acute kidney injury (AKI) is a common and serious problem. Globally, it has been estimated that one-in-five adult and one-in-three pediatric hospital inpatients suffer from AKI (1). AKI is characterized by kidney dysfunction and may occur secondary to ischemia or toxins or in the context of sepsis. Although this organ dysfunction is variably reversible, recurrent AKI may be associated with chronic damage to the kidneys, necessitating long-term renal replacement therapy with dialysis or transplantation. Altogether, this incurs significant health and economic costs (2), but there is currently no specific treatment for AKI.

Murine models of AKI have demonstrated a deleterious role for many immune cell subsets (3), including mononuclear phagocytes, NK cells, neutrophils, and T lymphocytes. However, B lymphocytes have received relatively little attention, although *μMt^−/−^* mice that are deficient in mature B cells have reduced severity of AKI following ischemia-reperfusion injury (4, 5) Other studies, however, suggest that B cells may also contribute to renal repair (6). B lymphocytes have previously been viewed primarily as Ab producers. However, there is increasing evidence that B cells have a much broader immunological remit as cytokine and chemokine producers that can both augment and inhibit inflammation, depending on the context (7). For example, IL-6, IFN-γ, and TNF-α from B cells can affect CD4 T cell behavior, whereas GM-CSF produced by so-called “innate response activator” B cells can stimulate neutrophil egress from the marrow (8). In contrast, regulatory B cells, through their provision of anti-inflammatory cytokines, such as IL-10 and IL-35, can inhibit autoimmunity in a range of conditions, such as colitis, collagen-induced arthritis, and experimental autoimmune encephalitis (9). In renal ischemia-reperfusion models, B cells have been identified as a source of CCL2, a monocyte-recruiting chemokine (10). Conversely, IL-10–producing regulatory B cells have been shown to attenuate AKI severity (11).

In this study, in murine models of AKI, we observed a 4-fold increase in circulating leukocytes within an hour of AKI induction, one third of which were B cells. There was a reduction in splenic B cells, suggesting that they mobilize into the circulation post-AKI, and a simultaneous increase in the number of B cells within the kidney parenchyma. Circulating B cells were phenotypically heterogeneous and included a CD19^+^B220^low^ subset that expressed CD11b. To further investigate the of role B cells in AKI in vivo, we used sialic acid–binding Ig-like lectin G (Siglec-G)–deficient mice with increased numbers of B220^low^ B cells and a lower threshold of B cell activation. *Siglecg*^−/−^ mice had more severe AKI compared with wild-type (WT) counterparts. During AKI, kidney B cells produced the chemokine CCL7, with the potential to facilitate neutrophil and monocyte recruitment. Indeed, blockade of CCL7 reduced both neutrophil and monocyte recruitment and ameliorated the severity of AKI in a murine model. In human kidneys with AKI, cellular deconvolution indicated an increase in B cells and in *CCL7* transcripts, and urine CCL7 levels were also elevated in patients with AKI. Together, our data suggest that B cells contribute to early sterile inflammation in AKI via the production of leukocyte-recruiting chemokines.

## Materials and Methods

All mice used in the experiments presented in this paper were drug or test naive and deemed to be healthy by animal facilities staff who regularly carried out health checks. All mice were single-sex-group housed following weaning, with standard food, temperature, and specific pathogen–free conditions approved by the U.K. Home Office. The experimental group was randomly assigned. All procedures were conducted in accordance with the U.K. Animals (Scientific Procedures) Act 1986.

### Folic acid–induced AKI

A 0.4-M sodium bicarbonate was made using stock 7.5% sodium bicarbonate solution (Sigma-Aldrich, Dorset, U.K.) and distilled water (produced in-house). Folic acid (Sigma-Aldrich) was dissolved in this solution to a concentration of 62.5 mg/ml and mixed on a rocker for 8 h at 36°C. Prior to injection, the folic acid (FA) was confirmed to be dissolved and was strained through a 40-μm filter to reduce the risk of bacterial contamination (Thermo Fisher Scientific, Waltham, MA). Mice were injected with FA i.p. with volumes calculated to be 100 μl for a 25 g mouse to achieve 250 mg/kg FA in all experiments, unless otherwise stated. In experiments lasting 15 h, FA injections were given at 17:00, and mice were sacrificed the following morning. In experiments that assessed earlier timepoints, FA was given at 09:00, and mice were sacrificed 3 or 6 h later. Prior to sacrifice, mice were housed at 36°C for ∼10 min before 100 μl of 3% anti-CD45 Ab was injected into the tail vein. Two minutes after the injection, mice were terminated using rising levels of carbon dioxide.

Kidneys were dissected and pushed through a 70-μl filter (Thermo Fisher Scientific) with 2 ml of digestion solution consisting of Collagenase D (1 mg/ml; Sigma-Aldrich), DNase I (0.1 mg/ml; Sigma-Aldrich), BSA (1%,; R&D Systems, Minneapolis, MN) in PBS at room temperature. A total of 5 × 10^4^ counting beads (Thermo Fisher Scientific, Waltham, MA) were placed into each sample. After 15 min, the samples were diluted in ice-cold complete RPMI 1640 medium (Sigma-Aldrich) containing BSA (1%) and penicillin/streptomycin (1 × 10^5^ U penicillin; Sigma-Aldrich) and placed on ice. Samples were washed, and red cell lysis solution was applied for 1 min. Cells were washed, blocked, Ab-stained, washed again, stained for viability, and fixed. Prior to analysis by flow cytometry, cells solutions were refiltered.

Blood was taken from by intracardiac puncture and stored in separating gel (Becton Dickinson, Franklin Lakes, NJ) or EDTA (Sarstedt, Newton, NC). Serum was centrifuged, extracted, and analyzed for urea by the Cambridge University Hospitals Core Biochemical Assay Laboratory. One hundred microliters of blood stored in EDTA was mixed with 1 ml of red cell lysis solution (produced in-house; 16.60 g of ammonium chloride, 2 g of sodium hydrogen carbonate, and 400 μl of 0.5 M EDTA [pH 8] in 2 l of Millipore water). Counting beads were placed into each sample. Cells were subsequently washed, blocked, Ab-stained, washed again, stained for viability, fixed, and analyzed as described below. A parametric, unpaired *t* test was used to test for statistical significance of serum urea levels.

Spleens were processed in a similar fashion to kidneys; however, in the place of digestion solution, complete RPMI 1640 was used when generating a cell solution.

A segment of femur was cut at the femoral neck and just proximal to the patellar surface. The marrow contents were flushed with 10 ml of ice-cold sterile PBS infused through a needle and syringe. Counting beads were not used for bone marrow analysis. Processing of the bone marrow followed the protocol used to process blood.

### Surgical renal pedicle clamping

Mice underwent laparotomy by a renal transplant surgeon or transplant surgical trainee with isoflurane general anesthetic, and renal hila were exposed. Fine surgical clips (Fine Science Tools, Foster City, CA) were placed over both renal hila to induce renal ischemia. After 45 min, the clips were removed, and reperfusion of the kidney was confirmed by changes to the color of the organ. The laparotomy wound was sutured, and mice were closely observed for signs of distress during recovery from the procedure both immediately and 4 h after the operation. Mice were sacrificed, and tissues were harvested after 24 h.

### Confocal microscopy of kidney

Kidneys were fixed in a buffer containing 1% paraformaldehyde (Electron Microscopy Services), l-lysine, and sodium periodate (both Sigma-Aldrich) for 24 h followed by 24 h in 30% sucrose (Sigma-Aldrich). Twenty-millimeter sections were permeabilized and blocked in 0.1 M Tris containing 0.1% Triton (Sigma-Aldrich), 10% normal mouse serum (made in-house), and 1% BSA. Images were acquired using an LSM 710 (Carl Zeiss, Oberkochen, Germany) confocal microscope. Abs were used at 1:100 concentration. Image analysis was done on Fiji/ImageJ. GR^+^ cells per visual field were counted. A parametric unpaired *t* test was used to test for statistical significance.

### H&E staining of samples and histological grading of AKI

Kidneys were fixed in formalin, embedded in paraffin, and cut into 7-μm sections before staining with H&E by Cambridge University Hospitals Core Biochemical Assay Laboratory. All analyses were performed blinded to sample identity. Tubulointerstitial damage was assessed by scoring three parameters: tubular necrosis, tubular dilatation, and cast formation. Scores were as follows: involvement of 0–25% of tubules within each cortical or medullary high-powered field, 1; 25–50%, 2; 50–75%, 3; and 75–100%, 4. Fifteen randomly chosen, nonoverlapping fields at ×400 magnification were blindly scored in each kidney section by a nephrologist trainee. A parametric unpaired *t* test was used to test for statistical significance.

### B cell isolation and stimulation

A splenocyte suspension was generated as described above. B cells were negatively isolated using magnetic bead selection, as per the manufacturer’s instructions, except the primary Ab-staining stage was extended to 15 min (Miltenyi Biotec, Bergisch Gladbach, Germany). Purity of B cells was consistently >95% when checked by flow cytometry. A total of 5 × 10^5^ B cells per well were cultured in 250 μl of complete RPMI 1640 supernatant. Cells were incubated at 36°C and 5% ambient CO_2_ for 48 h. B cells were stimulated with hen egg lysozyme (HEL; Roche, Basel, Switzerland) or CpG (ODN 2395; Miltenyi Biotec).

### ELISA

ELISAs measuring CCL7 of supernatant and urine were done as per the manufacturers’ instructions (human CCL7, R&D Systems; murine CCL7, Abcam, Cambridge, U.K.), except that all solution volumes were halved. Supernatants were analyzed in a 96-well plate (Nunc A/S, Kamstrup, Denmark) using a CLARIOstar Monochromator Microplate Reader (BMG Labtech, Ortenberg, Germany). After taking the arithmetic mean of replicates, a parametric unpaired *t* test was used to test for statistical significance.

### Human kidney microarray deconvolution analysis

Microarray gene expression data were obtained from the National Center for Biotechnology Information’s Gene Expression Omnibus through accession number GSE30718 (https://www.ncbi.nlm.nih.gov/geo/query/acc.cgi?acc=GSE30718). The dataset consisted of a cohort of 11 pristine biopsy specimens (no AKI) and 28 AKI biopsy specimens. The data were quantile normalized and Log2 transformed. Enrichment of immune cell populations was performed on this dataset with xCell (https://xcell.ucsf.edu/), using the xCell signature set (*n* = 64 cell types) (12). A Mann–Whitney *U* test and receiver–operating characteristic curve were performed using Prism software.

### Human kidney samples

Kidneys donated for transplantation but unsuitable for implantation because of damage to the arterial patch, parenchymal sclerosis, or suspicion of donor malignancy, were used. All analysis was performed in the U.K. Ethical approval was granted by the local ethics committee (REC12/EE/0446), and the study was also approved by National Health Service Blood and Transplant. Demographic donor data were retrieved from the National Health Service Blood and Transplant Electronic Offering System files. Patients with normal preterminal serum creatinine and not on renal replacement therapy were deemed to not have AKI. Patients with serum creatinine above 150 μmol/l were classified as having AKI. Samples were analyzed by quantitative PCR (qPCR) as described below. Statistical significance was assessed using a nonparametric Mann–Whitney *U* test, in which *p* < 0.05 was considered significant.

### qPCR

Whole murine kidneys and snap-frozen human kidney portions were placed in a tissue homogenizer (Bertin Instruments, Montigny-le-Bretonneux, France) with 1 ml of cell lysis buffer (Invitrogen, Carlsbad, CA). RNA was extracted using a preoptimized RNA purification set (QIAGEN, Hilden, Germany) as per the manufacturer’s instructions. The RNA yield was measured using a spectrophotometer (Thermo Fisher Scientific). Concentrations were standardized throughout each experiment by dilution with Nuclease-Free Water (Life Technologies, Carlsbad, CA). RNA underwent reverse transcription to cDNA as per the manufacturer’s instructions (Applied Biosystems, Foster City, CA; Bio-Rad Laboratories, Hercules, CA). Each sample was analyzed by PCR in triplicate using TaqMan reagents (Thermo Fisher Scientific) as per the manufacturer’s instructions. A parametric unpaired *t* test was used to test for statistical significance.

### Human AKI urine samples

Following ethical approval, patients who had no AKI or who had AKI were identified by a renal physician. Informed consent was taken, and urine was collected along with patient details, including age, ethnicity, baseline and acute creatinine, baseline and acute urea, standard urinalysis, medications, and comorbidities. Prior to analysis, samples were frozen in liquid nitrogen. Samples were defrosted at room temperature and analyzed by ELISA as described above. A parametric unpaired *t* test was used to test for statistical significance.

### Bone marrow and blood neutrophils

Eleven female WT C57BL/6 mice (Charles River Laboratories, Margate, U.K.) between 8 and 12 wk old were given between 50 and 250 mg/kg FA used to create FA-induced AKI (FA-AKI). Two blood samples clotted and therefore were not analyzed. In this experiment, an intravascular anti-CD45 Ab was not injected. Neutrophils were gated as single CD45^+^, GR1^+^, CD11b^+^ events. A parametric unpaired *t* test was used to test for statistical significance for this experiment and all other flow cytometry experiments described throughout this paper.

### Kidney neutrophils

Thirteen male WT C57BL/6 mice between 8 and 12 wk old were given between 50 and 250 mg/kg FA-AKI. One kidney was lost during processing for technical reasons. Neutrophils were gated as single in vitro CD45^+^, intravascular CD45^−^, GR1^+^, CD11b^+^ events.

### Global leukocyte numbers during the first 15 h of AKI

Nineteen male and female WT C57BL/6 mice between 7 and 16 wk old were given FA-AKI in two separate experiments. The 3 and 6 h time point experiment was conducted separately. Six, three, three, and seven mice were sacrificed at 0, 3, 6, 15 h, respectively, after FA-AKI induction. Splenic (a), blood (b), and kidney neutrophils (c) were gated as a) single in vitro CD45^+^, GR1^+^, CD11b^+^ events; b) single in vitro CD45^+^, GR1^+^, CD11b^+^ events; and c) single in vitro CD45^+^, intravascular CD45^−^, GR1^+^, CD11b^+^ events, respectively. B cells were gated as a) single, live, in vitro CD45^+^, CD19^+^ events; b) single, live, in vitro CD45^+^, CD19^+^ events; and c) single, live, in vitro CD45^+^, intravascular CD45^−^, CD19^+^ events, respectively. T cells were gated as a) single, live, in vitro CD45^+^, CD3^+^ events; b) single, live, in vitro CD45^+^, CD3^+^ events; and c) single, live, in vitro CD45^+^, intravascular CD45^−^, CD3^+^ events, respectively. Monocytes were gated as a) single, live, in vitro CD45^+^, GR1^−^, Ly-6c^+^ events; b) single, live, in vitro CD45^+^, GR1^−^, Ly-6c^+^ events; and c) single, live, in vitro CD45^+^, intravascular CD45^−^, GR1^−^, Ly-6c^+^ events, respectively.

### CD19^+^B220^low^ CD19^+^ B cells following FA-AKI

Thirteen male WT C57BL/6 mice between 10 and 12 wk old were given FA or vehicle control. CD19^+^B220^low^ CD19^+^ B cells were derived from single, live, in vitro CD45^+^ events.

### *Siglecg^−/−^* FA-AKI

Four male WT BALB/c and seven male *Siglecg^−/−^* (Siglecg^tm1Lnit^, MGI:3718495) mice from two littermate sets between 16 and 18 wk old received half-dose FA-AKI (125 mg/kg FA). Extravascular kidney B cells were gated as single, live, in vitro CD45^+^, intravascular CD45^−^, CD19^+^ events. Extravascular kidney neutrophils were gated as single in vitro CD45^+^, intravascular CD45^−^, GR1^+^, CD11b^+^ events. Extravascular kidney monocytes were gated as single, live, in vitro CD45^+^, intravascular CD45^−^, Ly-6C^low^MHCII^high^ events.

### *Siglecg^−/−^* cisplatin-induced AKI

Four male WT BALB/c and four male *Siglecg^−/−^* mice received 25 mg/kg cisplatin-induced AKI.

### *Siglecg^−/−^* surgical renal pedicle clamping AKI

Three male WT BALB/c and four male *Siglecg^−/−^* mice from three litters between 9 and 13 wk underwent surgical renal pedicle clamping.

### μMt mice whole kidney qPCR

Three 8–10-wk-old male WT C57BL/6 mice and three 8-wk-old littermate male μMt (B6.129S2-*Ighm*^tm1Cgn^/J) mice received FA-AKI.

### *Siglecg^−/−^* whole kidney qPCR

Five WT and four male *Siglecg^−/−^* from two litters received 125mg/kg FA-AKI. The mice were 12–14 wk old.

### WT B cell–stimulation assays

Negatively isolated splenic B cells from three 31-wk-old WT littermate female mice were stimulated with CpG at 5 μg/ml for 48 h.

### MD4 B cell–stimulation assays

Negatively isolated splenic B cells from three 8-wk-old heterozygous MD4 littermate male mice were stimulated with HEL at 10 ng/ml for 48 h.

### Anti-CCL7 FA-AKI

Mice were injected with 100 μl of PBS control i.p. or 20 μg of anti-CCL7 Ab in the same volume i.p. 4 h before injection of FA. Two identical experiments were combined. Two mice were untreated, nine received FA-AKI with isotype control injection, and five received FA-AKI with anti-CCL7 Ab. All mice were 12–14-wk-old WT C57BL/6 males. Gating occurred as described above in Global leukocyte numbers during the first 15 h of AKI.

### Data analysis

All data analysis was conducted on Fiji/ImageJ, FlowJo (version 10), GraphPad Prism (version 7.04), and Microsoft Excel (2019).

## Results

### Leukocyte dynamics in AKI

**FIGURE 1.**
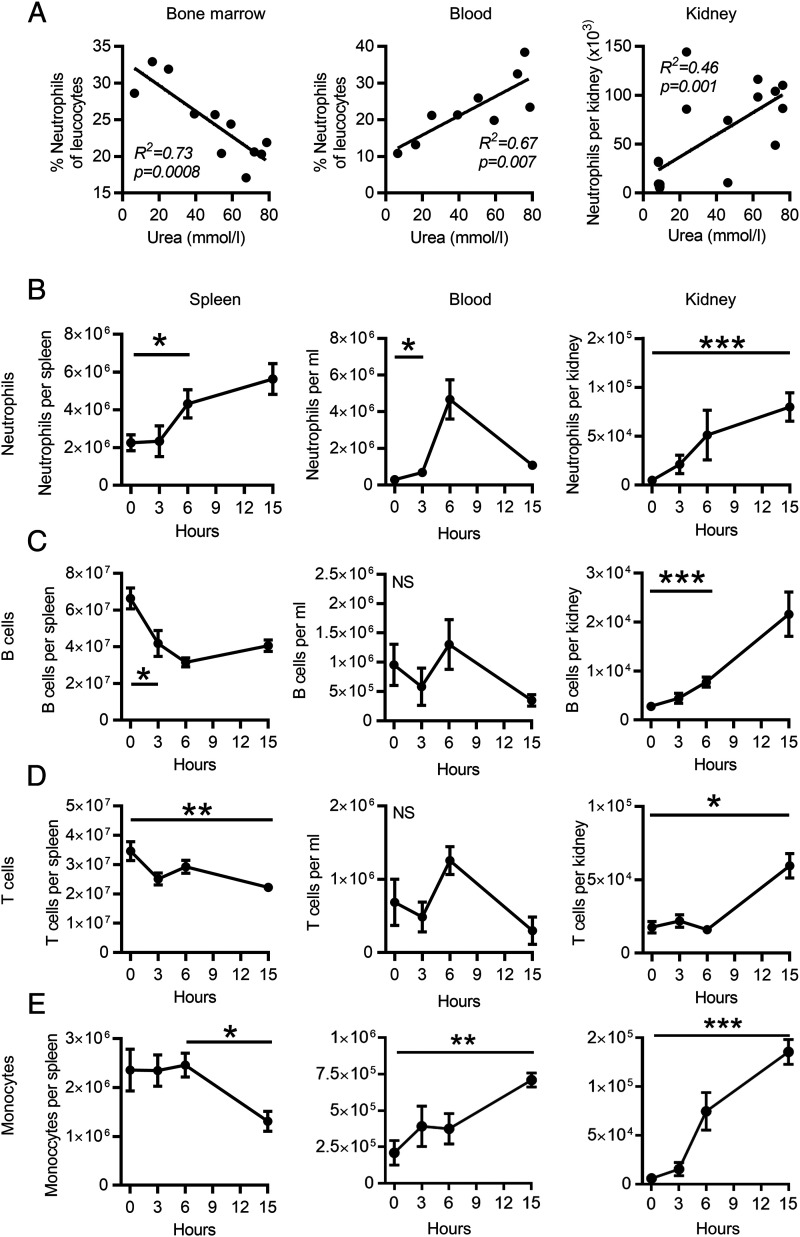
Leukocyte mobilization in the first 15 h following AKI. (**A**) Severity of AKI as measured by serum urea plotted against neutrophils in the bone marrow, blood, and kidney. All tissue was analyzed at 15 h. *R^2^* represents the quality of fit, and the *p* value tests the null hypothesis that the slope is equal to zero. (*n* = 11) female mice in experiment analyzing marrow and blood. (*n* = 13) male mice in experiment analyzing kidney neutrophils. AKI was induced by FA throughout this paper, unless stated otherwise. (**B**–**E**) Neutrophil, B cell, T cell, and monocyte numbers in the spleen, blood, and kidney in the first 15 h of AKI. Significance is indicated at the first time point at which *p* < 0.05 (*n* = 19 male and female mice) by parametric unpaired *t* test; error bars indicate SEM in all figures throughout this paper unless otherwise stated. *p < 0.05, **p < 0.01, ***p < 0.001, ****p < 0.0001.

We investigated the dynamics of leukocyte populations in lymphoid organs, blood, and kidney during the first few hours of AKI. Extravascular kidney leukocytes were identified by administration of a CD45 Ab i.v. immediately prior to organ harvest, as described previously (13). Following FA-AKI (14), neutrophil numbers decreased in bone marrow, with a reciprocal increase observed in blood and in the extravascular compartment of the kidney (Fig. 1A). Neutrophil numbers positively correlated with the severity of AKI, as indicated by elevated serum urea [bone marrow, *p* = 0.0008; blood, *p* = 0.007; kidney, *p* = 0.001; (Fig. 1A)]. B and T lymphocyte numbers in the spleen decreased in the first 3–6 h post-AKI, (Fig. 1C, 1D). In the case of B cells, this was mirrored by their increased infiltration into the kidney parenchyma (Fig. 1C). At a later time point (15 h), there was also an increase in kidney T cells and monocytes (Fig. 1D, 1E). These data suggest that leukocytes, including B cells, may mobilize from spleen in response to kidney injury and that neutrophils, monocytes, and B cells form an early immune infiltrate into the kidney in the first few hours following AKI.

### Increased in B cells in peripheral blood during AKI

**FIGURE 2.**
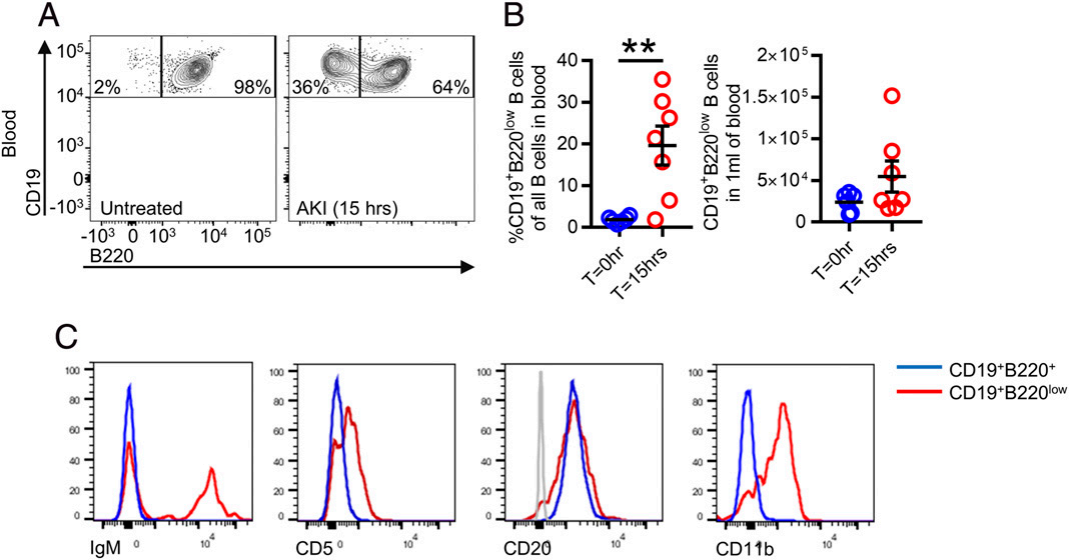
During AKI, a population of CD19^+^B220^low^ B cells is observed in the blood. (**A**) Representative flow cytometry chart of blood for healthy and AKI mice, gated on single, live, in vitro CD45^+^CD19^+^ events. (**B**) Quantification of percentage of CD19^+^B220^low^ B cells relative to all CD19^+^ B cells (left) and absolute number of CD19^+^B220^low^ B cells per 1 ml of blood in healthy mice and 15 h after FA-AKI mice. Right, (*n* = 13) male mice in total; parametric unpaired *t* test was used. (**C**) Representative histograms of other common B cells markers on blood CD19^+^B220^+^ and CD19^+^B220^low^ B cells 15 h following FA-AKI. Gray histogram in CD20 graph represents a negative population. **p < 0.01.

To further characterize the B cell response post-AKI, we performed flow cytometric analysis of peripheral blood B cells. This demonstrated the presence of more CD19^+^B220^low^ B cells in the blood following AKI induction, comprising around 20% of the circulating B cell pool (Fig. 2A, 2B). In contrast, this B cell subset was almost undetectable in the absence of AKI (Fig. 2A, 2B). CD19^+^B220^low^ B cells expressed comparable levels of CD20 to their B220^+^ counterparts but had higher surface expression of CD11b and CD5 (Fig. 2C), the latter a marker associated with B1a cells (15). Some of these cells were also IgM^+^, consistent with a B1a cell phenotype (Fig. 2C). They did not express CD3, Ly-6c, Ly-6g, and CD11c (Supplemental Fig. 1), confirming their identity as B cells.

### Mice with increased B1a cells have more severe AKI

**FIGURE 3.**
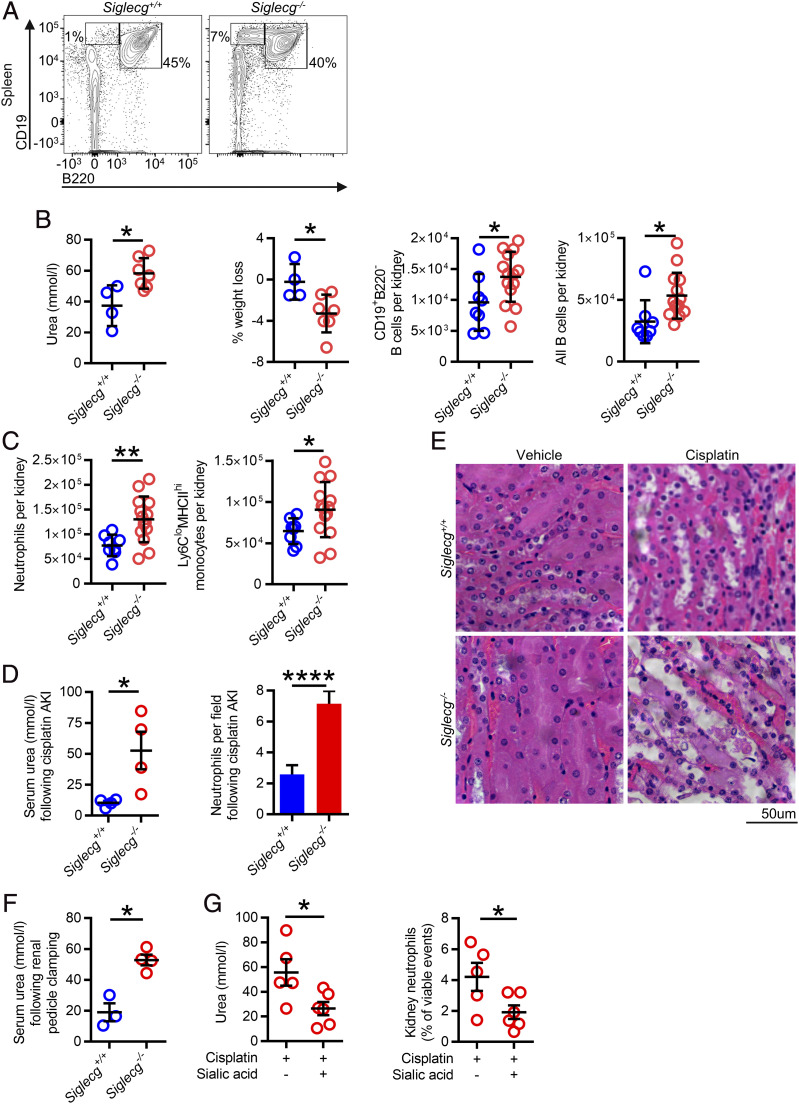
*Siglecg^−/−^* mice, which have excess B1a cells and B cell overactivation, have an exaggerated AKI immune response. (**A**) Representative flow cytometry plot showing WT and *Siglecg^−/−^* spleen CD19^+^B220^low^ B cells. (**B**) Serum urea, weight loss, and extravascular kidney B cells 15 h following FA-AKI in WT and *Siglecg^−/−^* mice (*n* = 11 male mice in total). A parametric unpaired *t* test was used. (**C**) Extravascular kidney neutrophils and monocytes following FA-AKI, as described in (B). (**D**) Serum urea and neutrophil count following cisplatin-induced AKI (*n* = 8 male mice in total); a parametric unpaired *t* test was used. (**E**) Representative H&E histological images of renal tubules following cisplatin-induced AKI. (**F**) Serum urea 15 h following surgical renal pedicle clamping–induced AKI (*n* = 7 male mice in total). A parametric unpaired *t* test was used. (**G**) Serum urea and kidney neutrophils following sialic acid–treated cisplatin-induced AKI in WT mice (*n* = 11 male mice in total). A parametric unpaired *t* test was used. *p < 0.05, **p < 0.01, ****p < 0.0001.

Siglec-G is an inhibitory receptor that is highly expressed on B cells and to a lesser extent on dendritic cells (data not shown). *Siglecg*^−/−^ mice are known to have higher number of B1a cells and a lower B cell activation threshold (16, 17). In keeping with the former observation, we found an increase in CD19^+^B220^low^ B cells in the spleen of *Siglecg*^−/−^ mice following AKI compared with their WT counterparts (Fig. 3A). We therefore used this strain to address the question of whether more readily activatable CD19^+^B220^low^ B cells might influence the course of AKI. Following induction of FA-AKI, *Siglecg*^−/−^ mice had increased numbers of total and CD19^+^B220^low^ B cells detectable in kidneys compared with WT mice (Fig. 3B). We observed increased severity of AKI in *Siglecg*^−/−^ mice, as evidenced by a significantly higher serum urea (mean 37.3 versus 58.2 mmol/l, *p* < 0.05), and greater weight loss, with increased neutrophil and monocyte infiltration into the kidney (Fig. 3C). To ensure general applicability of our findings, we used two additional models of AKI: cisplatin-induced AKI (Fig. 3D, 3E), a common iatrogenic cause of AKI in hospitals (18) and surgical clamping of the renal pedicle (Fig. 3F), modeling the prerenal hypoperfusion observed during transplantation or in the context of hemorrhage or hypotension. In both models, there was more severe AKI in *Siglecg^−/−^* animals.

Because Siglec-G may function to inhibit BCR-mediated activation (16, 17), we hypothesized that Siglec-G engagement using an agonist might inhibit AKI-associated B1a cell activation, reducing AKI severity. In keeping with this, administration of sialic acid Neu5Ac to WT mice led to reduced neutrophil recruitment and amelioration of AKI (Fig. 3G).

### B cells secrete CCL7, which exacerbates kidney injury

**FIGURE 4.**
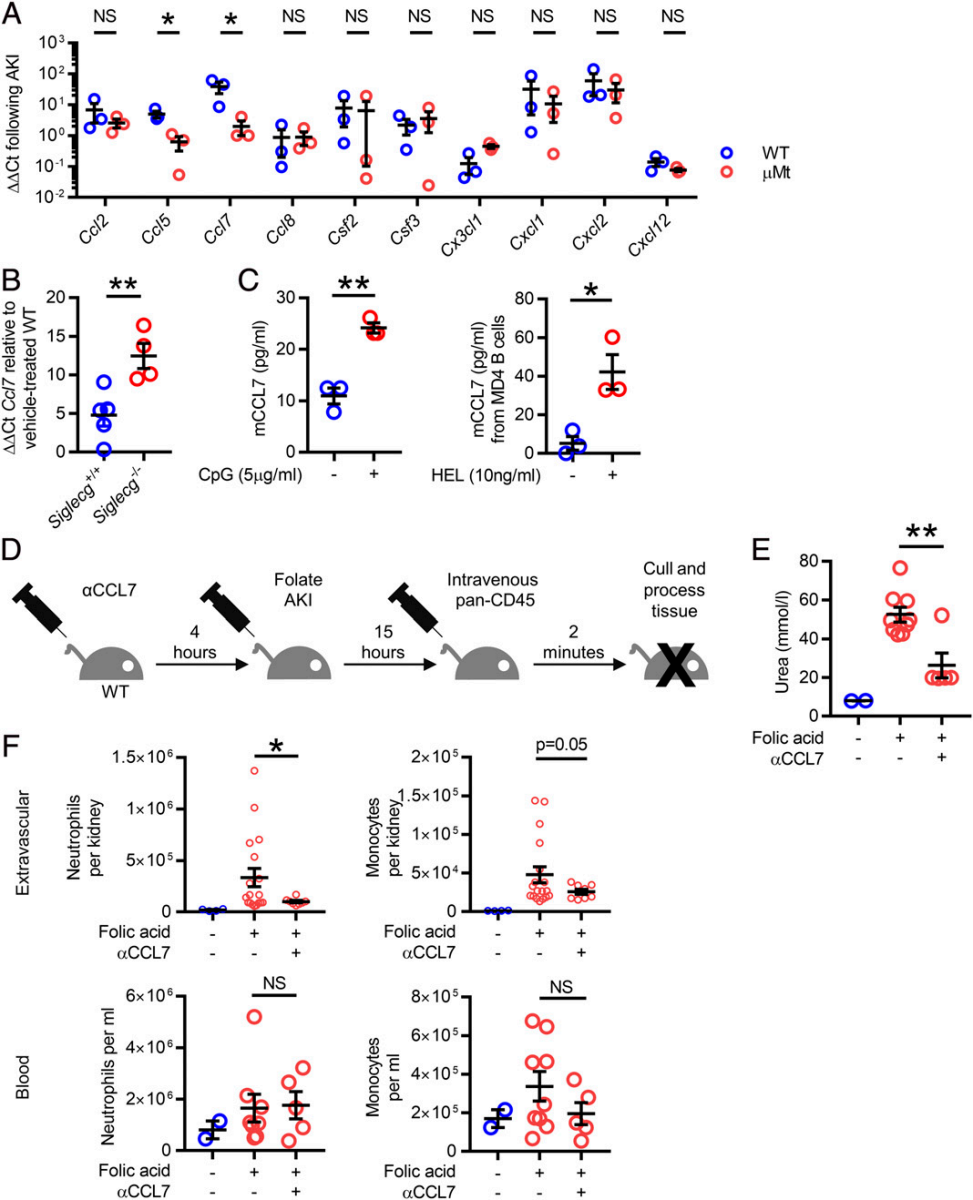
Provision of CCL7 from B cells attracts inflammatory myeloid cells into the kidneys during AKI. (**A**) qPCR from whole kidney lysate from WT and μMt mice (blue and red bars, respectively) 15 h following FA-AKI showing CCL5 and CCL7 significantly reduced in μMt mice (*n* = 6 male mice in total); a parametric unpaired *t* test was used. (**B**) *Ccl7* transcript levels in kidney lysate 15 h following FA-AKI in WT and *Siglecg^−/−^* mice (*n* = 9 male mice in total); a parametric unpaired *t* test was used. (**C**) Supernatant from negatively isolated WT B cells were measured for CCL7 by ELISA following stimulation with CpG (left). Supernatants from MD4 B cells were measured for CCL7 following BCR ligand stimulation with HEL (right, [*n* = 3 male mice in each experiment]; 5 × 10^5^ B cells stimulated for 48 h; a parametric unpaired *t* test was used.) (**D**). Schematic of experiment inhibiting CCL7 during AKI. (**E**) Serum urea following with CCL7 inhibition (*n* = 16 male mice in total); two identical experiments were combined; and a parametric unpaired *t* test was used. (**F**) Extravascular renal and blood neutrophils and monocytes following CCL7 inhibition. *p < 0.05, **p < 0.01.

When considering the mechanism by which B cells may influence AKI severity, we hypothesized that kidney B cells may secrete chemokines that might influence the recruitment of immune cells to the kidney during AKI. To test this, we assessed chemokine transcript levels in kidneys obtained from WT and *μMt^−/−^* mice following induction of AKI. We observed significantly lower levels of *Ccl5* and *Ccl7* in *μMt^−/−^* kidneys, whereas *Ccl2* transcripts were similar in WT and *μMt^−/−^* kidneys (Fig. 4A). Because *Ccl7* transcripts were substantially higher in WT kidneys than *Ccl5* transcripts, we chose to focus subsequent experiments on CCL7. In contrast to *μMt^−/−^* mice, kidney *Ccl7* transcripts were higher in Siglec-G–deficient mice than those observed in WT counterparts, in keeping with the conclusion that B cells are an important source of CCL7 during AKI (Fig. 4B). In vitro, splenic B cells secreted CCL7 following CpG stimulation and BCR cross-linking (Fig. 4C), confirming the capacity of B cells to produce CCL7 following activation in both an Ag-dependent and -independent manner. Of note, CCL7 can act as both a monocyte and neutrophil chemoattractant (19–22). Therefore, to understand whether kidney B cell–derived CCL7 may play a role in AKI by influencing myeloid cell recruitment, we administered a polyclonal CCL7-blocking Ab at the time of AKI induction (Fig. 4D). CCL7 inhibition ameliorated the severity of AKI, as evidenced by a reduction in serum urea and was associated with less neutrophil and monocyte infiltration into the kidney interstitium compared with isotype control-treated animals (Fig. 4E, 4F). There was no significant difference in the number of intravascular neutrophils and monocytes between CCL7 Ab and isotype control-treated animals (Fig. 4F).

### B cells and CCL7 associated with AKI severity in humans

**FIGURE 5.**
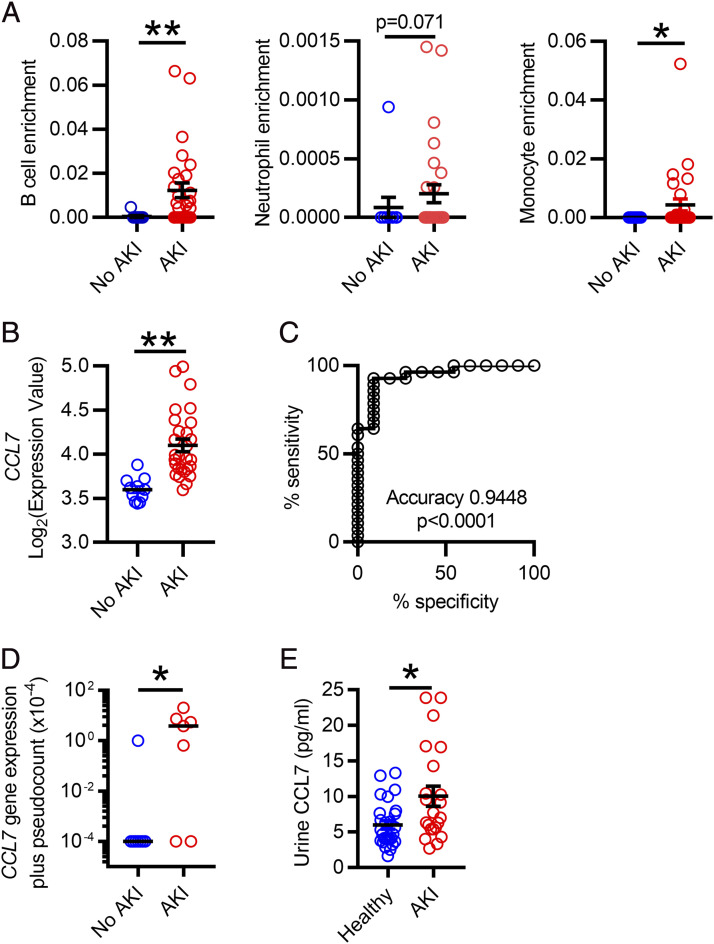
*CCL7* is expressed in human kidneys during AKI. (**A**) Deconvolution analysis of B cell, neutrophil, and monocyte enrichment from biopsy specimen–confirmed healthy and AKI kidneys of previously published dataset (*n* = 39 humans in total); a nonparametric Mann–Whitney *U* test was used. (**B**) *CCL7* transcript from the same dataset. (**C**) Receiver-operating characteristic curve of *CCL7* transcripts as a classifier of AKI from the same dataset. (**D**) *CCL7* qPCR of lysate from snap-frozen kidney cortex from donors with and without preterminal AKI (*n* = 15 humans in total); a parametric unpaired *t* test was used. (**E**) ELISA measurements of CCL7 in urine from patients with and without AKI (*n* = 49 humans in total); a parametric unpaired *t* test was used. *p < 0.05, **p < 0.01.

To assess the significance of these findings to human pathology, we analyzed a publicly available microRNA dataset generated from 39 kidney biopsy specimens (11 normal and 28 AKI biopsy specimens) (23). Cellular deconvolution revealed enrichment of B cells, neutrophils, and monocytes in human kidneys with AKI, with no significant change in CD4 and CD8 T cells (Fig. 5A, Supplemental Fig. 2). Furthermore, *CCL7* transcripts were predictive of AKI, when used as a binary classifier (Fig. 5B, 5C). To confirm the presence of elevated CCL7 within AKI kidneys and to validate our findings in a second cohort, we assessed samples from kidney organ donors with normal and abnormal creatinine levels at the time of retrieval (for baseline characteristics, see Supplemental Table I). We observed significantly higher levels of *CCL7* transcript in kidneys with AKI than those with normal renal function (Fig. 5D). In addition, in a further, independent cohort of patients with AKI, urine CCL7 was significantly higher in AKI urine compared with that obtained from control subjects with normal kidney function (Fig. 5E, Supplemental Table II).

## Discussion

Previous studies have described an expansion of lymphocytes, including T cells, in the kidney during AKI because of both migration and local proliferation (3, 24). Our study did not specifically address the relative contribution of migration versus in situ proliferation in B cells, but it is likely that an influx from the substantially expanded circulating B cell pool makes a contribution. In our study, we show that circulating B cell populations change within hours of induction of AKI. These include a B220^low^ subset, some of which expressed CD5, consistent with an innate B1a identity. Of note, TLR stimulation may lead to downregulation of CD5 expression on B1a cells (25). Therefore, the mixed phenotype we observed in the B220^low^ subset may be related to exposure to TLR-engaging circulating damage-associated molecular patterns associated with AKI. Some of these B220^low^ cells were IgM^−^, which is atypical for B1 cells, and all B220^low^ cells expressed CD11b. CD11b forms the MAC-1 integrin complex along with CD18, and its expression is typically observed on myeloid cells. MAC-1 enables cellular interactions with ICAM1 to facilitate endothelial cell adhesion. Of note, CD11b^+^ B cells have previously been described in mice with experimental autoimmune hepatitis (26), and in the lung, type 1 IFNs were shown to activate CD11b on B1 cells, enabling their entry into mediastinal lymph nodes in the context of influenza infection (27). Therefore, in the context of AKI, CD11b expression on B cells may play some role in cell migration.

We found that kidney B cells are an important source of CCL7, a chemokine that functions as an attractant for both neutrophils and monocytes (19–22). In our AKI model, kidney *Ccl2* transcripts were similar in WT and B cell–deficient mice, suggesting that B cells were not a major source of this monocyte-recruiting chemokine, in contrast to a previous study (10). This difference may relate to the ureteric obstruction model Han et al. (10) used in contrast to our FA model. Notably, the production of CCL7 by B cells has been described in sterile inflammation in other contexts, for example, ischemia-reperfusion injury in myocardial infarction (28). Although our study did not definitively test the exclusive importance of B cell–derived CCL7, inhibition of this cytokine significantly ameliorated AKI, with the greatest effects observed on neutrophil rather than monocyte recruitment (Fig. 4E). It is possible that other cells, both lymphoid and myeloid, may make a contribution to the production of CCL7 in the injured kidney. Although CCL7 can function to mobilize myeloid cells from the bone marrow (21), its major effect in AKI is likely to be the attraction of monocytes and neutrophils to the kidney rather than inducing bone marrow egress, given that peripheral neutrophil counts were largely unchanged between mice treated with the CCL7 blocking Ab compared with controls (Fig. 4F).

To confirm the importance of our observations to human AKI, we analyzed *CCL7* transcripts in the kidney and CCL7 protein in the urine, and both were significantly elevated in patients with AKI. These observations have two implications. First, urinary CCL7 could represent a useful biomarker for AKI, although its sensitivity and specificity would need to be tested in a much larger sample set. Second, the murine data suggest that CCL7-blockade may be a useful therapeutic strategy to reduce inflammatory cell infiltration into the kidneys, thereby ameliorating AKI, without affecting systemic mobilization of myeloid cells that may be helpful for defense against infections. In keeping with our data, one previous study demonstrated that *Ccl7*-deficient mice showed attenuated inflammatory cell recruitment and tubulointerstitial fibrosis at early timepoints after ureteric obstruction (29), but, as far as we are aware, our data are the first to investigate CCL7 in human samples in AKI.

Because B cells represent a significant source of CCL7, and our observations in *Siglecg^−/−^* mice, would suggest B1a cells in particular, targeting this cell subset particularly may be of utility. In this regard, Siglec-G itself (or its human ortholog SIGLEC-10), may represent a direct target: Siglec-G is a surface glycoprotein that has an ITIM in its cytoplasmic domain, enabling it to function to inhibit BCR-mediated activation (16, 17). Therefore, Siglec-G engagement using an agonist, such as sialic acid, may hold therapeutic potential. Indeed, previous work in a rat model has shown that concomitant administration of sialic acid can improve outcomes from AKI, although a wholly different mechanism was proposed to that we have demonstrated (30). One issue with inhibiting B1a function is that this subset may also be an important source of the immunoregulatory cytokine IL-10 (31). Therefore, inhibiting this subset may be undesirable, given potential beneficial effects of regulatory B cells in models of stroke (32) and AKI (11).

Previous studies have shown sex-based differences in severity of AKI (33–36) With the exception of one set of experiments, all in vivo murine AKI experiments used only male mice. Although the murine experiments were conducted primarily in males, the overall findings are likely to be applicable across sexes, given that our data from human kidneys and urine (Fig. 5D, 5E) included samples from both males and females (Supplemental Tables I, II).

In summary, we have identified that kidney B cells are an important source of CCL7 during AKI, promoting neutrophil and monocyte recruitment to exacerbate AKI severity. Our data indicate that CCL7 warrants further investigation as both a biomarker and therapeutic target in AKI.

## Supplementary Material

Data Supplement
