# Supplementary material for: B Lymphocyte–Derived CCL7 Augments Neutrophil and Monocyte Recruitment, Exacerbating Acute Kidney Injury
Source: J Immunol. 2020 Jul 31;205(5):1376–84. doi: 10.4049/jimmunol.2000454 (PMC7444279; doi:10.4049/jimmunol.2000454)
Supplement: Data Supplement [file JI_2000454.zip › JI_2000454_Supplemental_Material_1.pdf]

Supplementary figures and tables

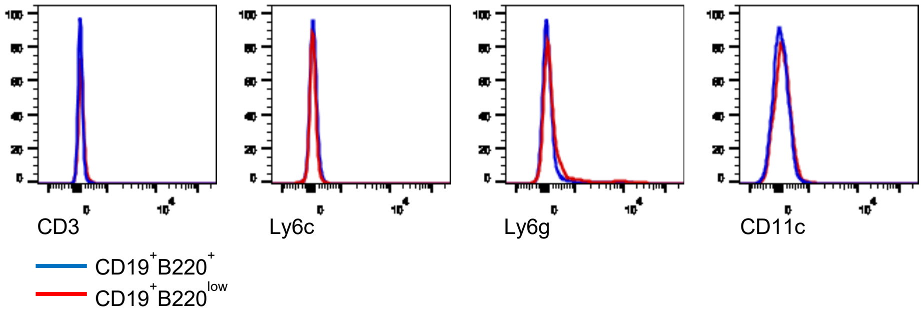

Figure S1: Representative histograms of non-B cells markers on blood CD19^{+}B220^{+} and CD19^{+}B220^{low} B cells.

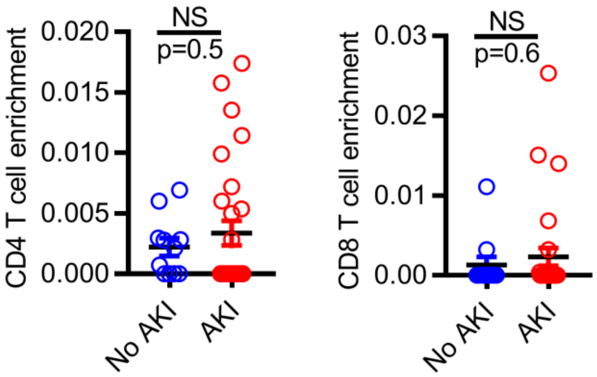

Figure S2: Deconvolution results of CD4 and CD8 T cell type enrichment analysis using xCell and xCell signature set on microarray gene expression data from biopsy-confirmed healthy (blue; n=11) and AKI (red; n=28) kidneys derived from GSE30718. The y-axis shows the enrichment score in each sample (n=39 humans in total). Statistical significance was assessed using a non-parametric Mann-Whitney U test where $p < 0.05$ was considered significant. NS denotes not significant.

Table S1: Summary of cadaveric donor demographic details

	No AKI	AKI	p value
**N**	8	7	N/A
**Preterminal serum creatinine**	48.5 ± 2.9 μmol/L	195.6 ± 14.7 μmol/L	<0.001
**Age**	66.4 ± 3.8 years	59.86 ± 4.7 years	0.295
**Sex**	4 males<br>4 females	4 males<br>3 females	0.14<br>(chi-squared)

[1]

[1] **Table S1**: Summary of cadaveric donor demographic details.

Table S2: Demographic summary of patients with AKI whose urine was analysed for CCL7 protein levels.

	**No AKI**	**AKI**	**p value**
**n**	27	22	NA
**Serum creatinine**	69.1 ± 4.3 µmol/L	337.8 ± 73.6 µmol/L	<0.0001
**Age**	50.8 ± 10.2 years	72.2 ± 3.7 years	0.023
**Sex**	13 males<br>14 females	12 males<br>10 females	0.66<br>(chi-squared)

[2]

[2] **Table S2:** Demographic summary of patients with AKI whose urine was analysed for CCL7 protein levels.
